# No one is better than all together: the role of networks in pediatric intensive care

**DOI:** 10.5935/0103-507X.20190043

**Published:** 2019

**Authors:** Sebastián González-Dambrauskas, Juan Camilo Jaramillo-Bustamante, Franco Díaz

**Affiliations:** 1 Red Colaborativa Pediátrica de Latinoamérica (LARed Network) - Montevidéu, Uruguai.; 2 Cuidados Intensivos Pediátricos Especializados, Casa de Galicia - Montevidéu, Uruguai.; 3 Unidad de Cuidados Intensivos Pediátricos, Hospital General de Medellín - Medelín, Colômbia.; 4 Cátedra de Facultad de Medicina, Universidad de Antioquia - Antioquia, Colômbia.; 5 Facultad de Medicina Clínica Alemana, Universidad del Desarrollo - Santiago, Chile.; 6 Unidad de Cuidados Intensivos Pediátricos, Clínica Alemana de Santiago - Santiago, Chile.

## INTRODUCTION

Pediatric critical illness is a rare event. Crow et al. recently showed how unusual it is for a child to be admitted to a pediatric intensive care unit (PICU).^(1)^ Although infrequent from a population-based standpoint, common PICU conditions (such as sepsis) represent an international threat, and significant disparities in outcomes still prevail in resource-limited settings.^(2,3)^

Children hospitalized in PICUs have unique characteristics, heterogeneous diagnoses and a wide range of ages. Furthermore, many pragmatic obstacles, such as substantial variation in the process of care delivery across different healthcare systems (hospital organizational differences, low access to home care services, low availability of beds, etc.), can coexist and make it difficult to obtain representative sample sizes for PICU research. Usually, a single PICU does not have enough patient volume to gather substantial amount of information about a specific homogeneous group of children to achieve good-quality research. Therefore, variability in common clinical practices among providers is widespread, which can become a driver for the misuse of therapeutic and diagnostic interventions and an obstacle to improving health-care outcomes.

Fortunately, in the last few years, many collective efforts have been made to successfully move the field on PICU research forward using the research network model. This article provides brief insights into research networks, with a special focus on collaborative networks.

## NETWORKS IN THE ERA OF THE SUSTAINABLE DEVELOPMENT GOALS. IMPROVING THE VALUE OF CARE AND RESEARCH

Several national, regional, and international-based PICU research groups and formal consortiums have started standardized registries and, in some cases, have adopted the network model to conduct research worldwide. A list of established and currently active groups is displayed in table 1.

**Table 1:** Established (up to March 2019) and active pediatric intensive care unit networks/consortiums. Disease-specific and therapy-specific groups were excluded

Name	Main focus	Type of PICU/Location	Website
Australian and New Zealand Intensive Care Society Paediatric Study Group (ANZICS-PSG)	Clinical research	10 PICUs (and an additional 20 ICUs that treat children and send data)/Australia & New Zealand	https://www.anzics.com.au/paediatric-study-group/
Australian and New Zealand Intensive Care Paediatric Registry (ANZPIC-R)	Registry	10 PICUs (and an additional 20 ICUs that treat children and send data)/Australia & New Zealand	https://www.anzics.com.au/australian-and-new-zealand-paediatric-intensive-care-registry-anzpicr/
Brazilian Research Network in Pediatric Intensive Care (BRnet-PIC)*	Clinical research	52 PICUs/Brazil	N/A
Canadian Critical Care Trials Group (CCTG)	Clinical research	Investigator-driven	https://www.ccctg.ca/CCCTG/Pediatric-Interest-Group.aspx
Collaborative Pediatric Critical Care Research Network (CPCCRN)	Clinical research	7 PICUs/United States of America	https://www.cpccrn.org/
Italian Pediatric Intensive Care Units Network (TIPNet)	Registry	18 PICUs/Italy	http://tipnet.cineca.it/index.php
LARed Network (LARed)*	Registry/clinical research	35 PICUs/8 Latin American countries	https://www.la-red.net
Paediatric Intensive Care Audit Network (PICANet)	Registry	35 PICUs/Ireland, United Kingdom	https://www.picanet.org.uk/
Pediatric Intensive Care Evaluation (PICE)	Registry	7 PICUs/Netherlands	https://pice.nl/
Paediatric Critical Care Research Group (PCCRG)	Clinical research	7 PICUs/Brisbane Region, Australia	N/A
Pediatric Acute Lung Injury and Sepsis Investigators (PALISI)	Clinical research	90 + PICUs/United States of America and Canada	https://www.palisi.org/
Sociedad Argentina de Terapia Argentina Quality (SATIQ) *	Registry/clinical research	49 PICUs/Argentina	https://www.facebook.com/groups/satiq/ http://www.hardineros.com.ar/satiq/
Virtual Paediatric Intensive Care Unit Performance System (VPS)	Registry	100 + PICUs and PCICUs/United States of America	http://vpicu.net

ICU - intensive care unit; PCICU - pediatric cardiac intensive care unit; PICU - pediatric intensive care unit; N/A - not available

* Latin America-based groups.

These groups have different organizational characteristics (funding bodies and institutional partnerships, among others) and are primarily based in developed countries. Their common purpose has been to conduct clinical and translational research to better inform clinical PICU practice. They have designed and executed many PICU observational/interventional trials as well as feasibility studies that have allowed innovative proposals for new research agenda directions that are more focused on pragmatic, real-world translational clinical studies that could lead to increased generalizability of results.^(4)^

Pediatric intensive care networks have also used formal data sources for research. Many available large datasets have helped investigators increase the understanding of current clinical practice and provide data for trials.^(5)^ These datasets vary worldwide in the level of clinical detail, type of data (disease-specific and nonspecific registries, benchmarking databases), accessibility (free/paid), origin (public/private) and population representation (population-based registries or not).

**Figure 1:**
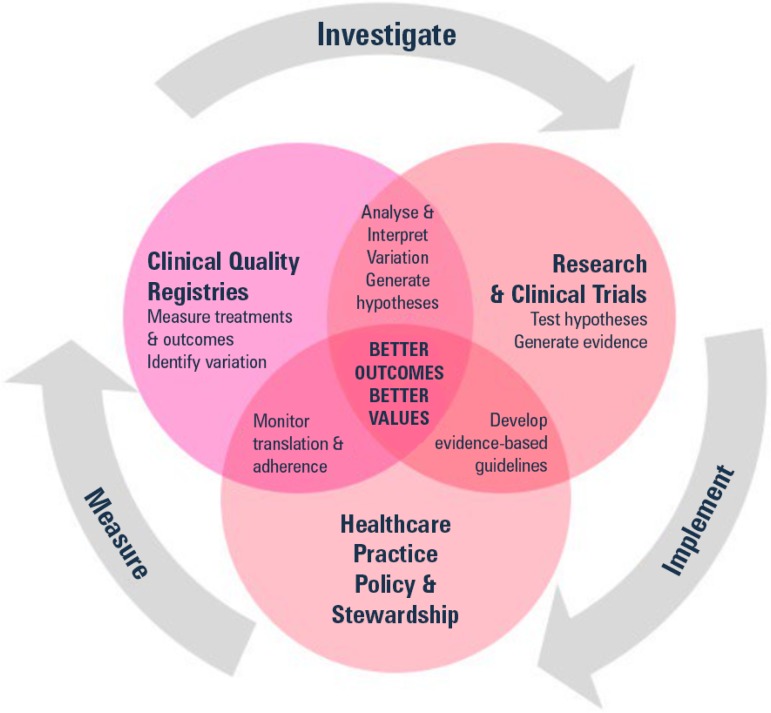
Self-improving healthcare system cycle. Evidence from investigator-initiated clinical trials by established research networks can fuel the development of evidence-based clinical guidelines and health policies, and then their implementation can be measured and analyzed by different quality registries (identifying variability in care and what works and does not work in everyday clinical practice). Finally, new research questions can arise from different stakeholders, thus generating new hypotheses to test in further clinical trials. This core function of the cycle may lead not only to improved clinical practice quality and outcomes but also to improved healthcare value.

Source: Extracted from Australian Clinical Trials Alliance. Economic evaluation of investigator-initiated clinical trials conducted by networks [Internet]. Sydney: ACSQHC; 2017. [cited 2019 Jul 18]. Available at: https://www.safetyandquality.gov.au/wp-content/uploads/2017/07/Economic-evaluation-of-investigator-initiated-clinical-trials-conducted-by-networks.pdf. Accessed October 9, 2018.^(6)^

The network model pays off. A recent report showed that investigator-initiated clinical trials conducted by established networks have a gross benefit of $2 billion dollars, based on better health outcomes and reduced healthcare service costs to the Australian economy.^(6)^ There is a remarkable return of $5.8 for every $1 invested by society in network trials. Compared with industry-led trials, research network trials are cheaper, can ensure a higher citation impact and eventually influence relevant clinical practice changes.^(7)^ As shown in figure 1, networks can become a core element of the quality improvement cycle for healthcare systems and enhance the use of evidence in practice through the self-improvement cycle.

Choong et al. reported that although clinical trials from PICU networks represent a minority of the trials ever conducted, PICU network trials are larger, target more clinically relevant outcomes, are more likely to be funded and have a greater citation impact than nonnetwork trials.^(8)^ These benefits of network trials have led to an overall perception among the PICU community that research networks are one of the available facilitators to overcome common barriers to conducting rigorous randomized controlled trials that are needed to inform clinical practice.^(9)^

## NETWORKS: IT IS NOT JUST ABOUT RESEARCH

Some networks have integrated a spirit of collaboration into their agendas and have focused not only on research but also on quality improvement initiatives. Lannon et al. defined pediatric collaborative networks (PCNs) as

multisite clinical networks that allow practice-based teams to learn from one another, test changes to improve quality, and use collective experience and data to understand and ultimately implement and spread what works in practice.^(10)^

As a result of their work, PCNs can identify the gaps between evidence and practice, as well as provide the tools to close those gaps and thus improve health outcomes. Remarkable examples of PCNs in the PICU field include pediatric cardiac critical care collaboratives and consortiums, which have already adopted a shared vision among clinicians to improve the value of care in these vulnerable populations.^(11)^

Usually, PCNs contribute to standardized data collection and have a strong commitment to sharing and using data to improve health care outcomes. As a core part of their work, PCNs construct robust multicentric databases with different cohort registries, which can easily overcome the inherent limitations of population data to obtain significant samples and increase statistical power. These data typically provide valuable information to members of the network through different high-quality performance reports, which gives network members the capacity to identify the actual opportunities for improvement, track network members' performance over time and conduct benchmarking processes and then generate the necessary information (metrics, quality clinical indicators, risk-adjusted outcomes) to inform clinical performance and allow comparison against the rest of the participants in the network. In PCNs, *real-world data* lead to action, and practice can become standardized, potentially reducing variability in care, avoiding common misuse practices and improving health system value.

Pediatric collaborative networks support quality improvement and research^(12)^ since they usually engage multiple essential stakeholders such as patients, families, advocacy organizations, policy makers, hospitals, clinicians and researchers. In 2013, the American Academy of Pediatrics published a supplement that outlined the reasons why PCNs have become a proven and transforming principle in pediatrics.^(13)^ Why? Because PCNs can work at most levels of clinical research science implementation, identifying translational patient-centered outcomes research ("the right treatment for the right patient in the right way at the right time") and creating tools to implement personalized changes.^(14)^

## FOCUS ON LATIN AMERICA

Latin America is a vast and diverse geopolitical region composed of 20 countries that contain mosaic of fragmented and diverse health care systems in which complex obstacles prevent improvement in the value of patient care. In this context, Brazil provided research networks with fertile ground for successful development and large, high-quality databases in adult critical care.^(15,16)^ BRICNet (Brazilian Research in Intensive Care Network) and LIVEN (Latin American Intensive Care Network) are great examples of two rapidly expanding and productive networks in Latin America. However, to our knowledge, there were no active PICU collaborative networks in the continent until recently. One of these networks is LARed (Table 1).

**Figure 2:**
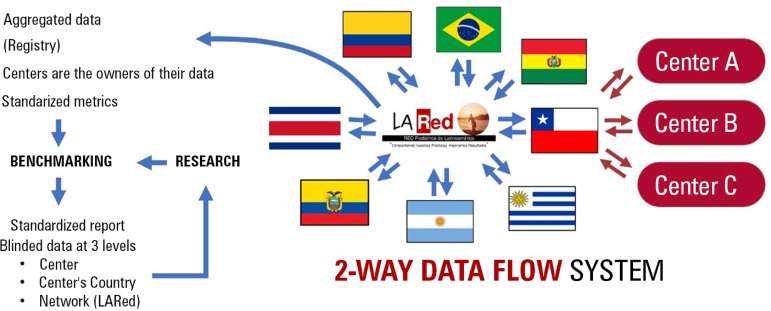
LARed database model using LARed-REP (the LARed online report system). In contrast to classic academic research models (one-way models), the system is a 2-way data flow model. The centers own their data and share it with local, regional and central databases. A deidentified and blinded aggregated data registry allows standardized metrics for benchmarking and, eventually, for research addressing heterogeneity and context. Each center can compare their performance on 3 levels (local, regional and global), with online, real-time feedback.

LARed Network (LARed: www.la-red.net) is a nonprofit, independent Latin American organization built by PICU professionals in 2014.^(17)^^)^ Inspired by the Vermont Oxford Neonatal Network experience, it has expanded to 8 countries (Argentina, Bolivia, Brazil, Chile, Colombia, Costa Rica, Ecuador and Uruguay), involving more than 35 PICUs. It has embraced the PCN model and fostered international collaboration, establishing an innovative registry/database (Figure 2). This unfunded, free-of-charge network is open to any PICUs from the region that are interested in joining. The process of on-boarding is easy, and all PICU members are invited to contribute data to the LARed registry; propose new studies; and participate in collaborative initiatives, as well as clinical, epidemiological and quality improvement studies. LARed is currently working on the development of many projects with other networks/registries around the globe and is an active participant of the World Federation of Pediatric Intensive and Critical Care Societies (WFPICCS).

The LARed registry has the potential to provide high-quality data that would allow us to detect variability in care, promote quality improvement initiatives and trigger real-world research questions. The data gathered have also led us to change our approach by adapting international guidelines, protocols and recommendations for what we call "context-based medicine". Narrowing the gap between evidence and practice has become an achievable goal for our heterogeneous region.

The culture of network collaboration is thriving worldwide. Networks might be the best available tool to transform the PICU field by achieving the much-needed excellence in clinical care that patients and families expect and deserve. Everyone is invited to join this transformative movement.
